# Supplementary material for: Genetic Variation in Complex Traits in Transgenic α-Synuclein Strains of Caenorhabditis elegans
Source: Genes (Basel). 2020 Jul 11;11(7):778. doi: 10.3390/genes11070778 (PMC7397059; doi:10.3390/genes11070778)
Supplement: Supplementary file 1 [file genes-11-00778-s001.zip › Figure S1-3.docx]

| A |
| --- |
| **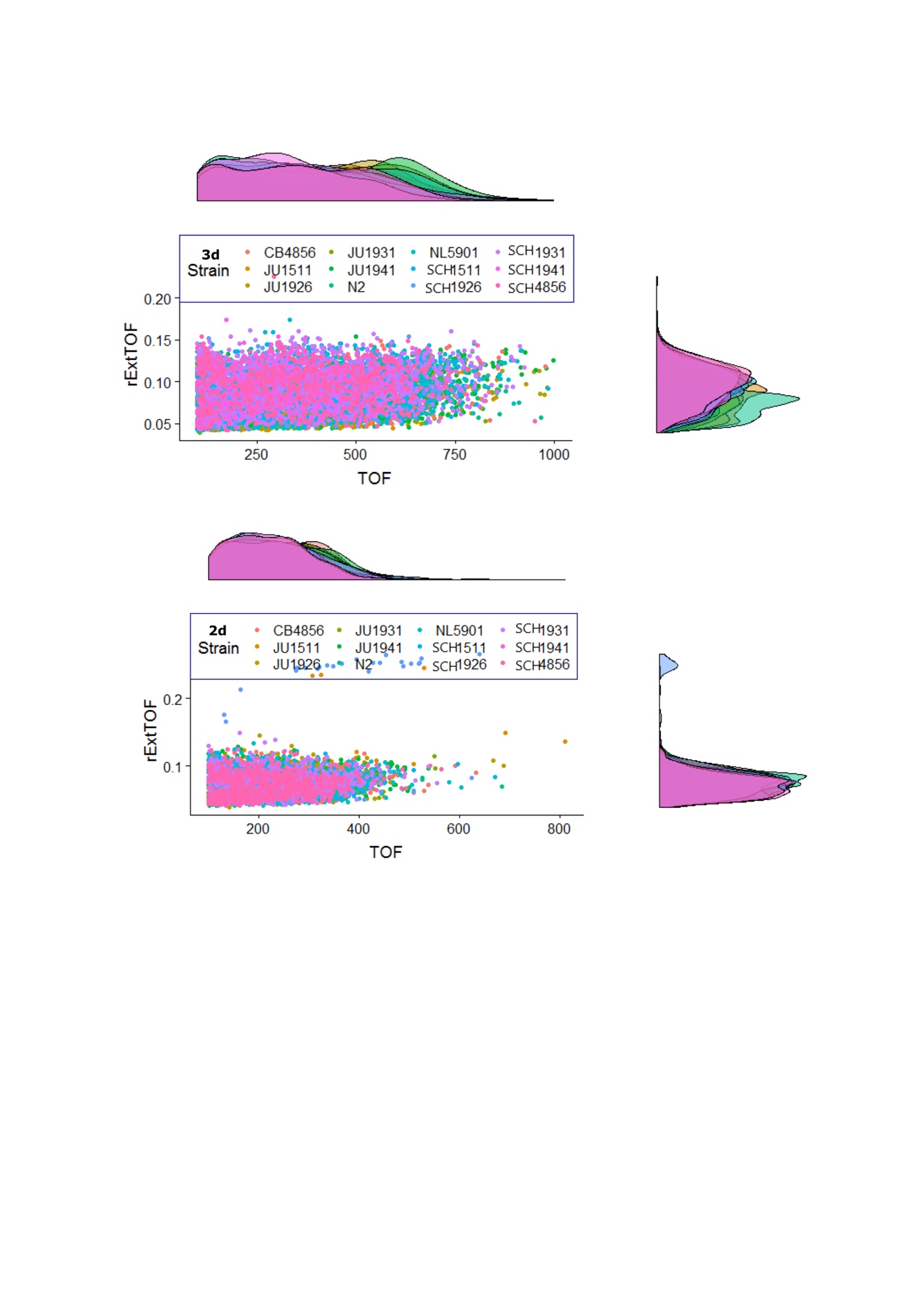**  **B** |
| **Figure S1.**  Normalization of worm body parameters with overall measurement of YFP fluorescence per strain per sample. The measurements were done with 3 days and 2 days old worms (A and B, respectively). The x-axis is the time-of-flight (TOF), while the y-axis is the ratio of Extinction by TOF (rExtTOF). Raw data of all 3 days old worms, which was collected from a Union Biometrica COPAS BIOSORT large particle flow, were filtered with both TOF (250, 1000) and rExtTOF (0.05, 0.15), while that of 2 days old was done with TOF (100, 500) and rExtTOF (0.05, 0.15). This filtering is for avoiding the technical noise. |

| **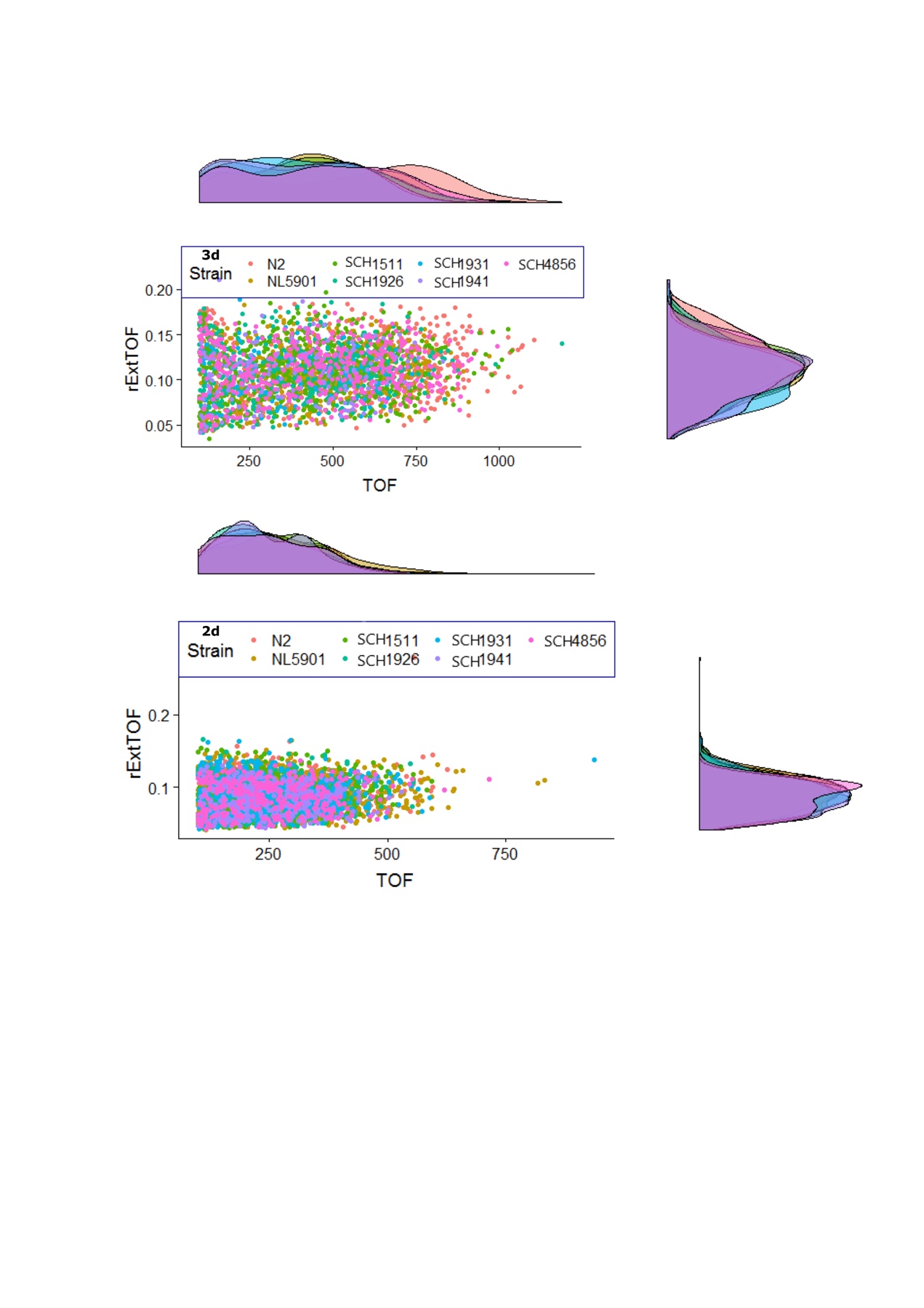**A |
| --- |
| **B** |
| **Figure S2.** Normalization of worm body parameters with overall measurement of Nile Red fluorescence per strain per sample. The measurements were done with 3 days and 2 days old worms (A and B, respectively). The x-axis is the time-of-flight (TOF), while the y-axis is the ratio of Extinction by TOF (rExtTOF). Raw data of all 3 days old worms, which was collected from a Union Biometrica BIOSORT large particle flow, were filtered with both TOF (250, 1000) and rExtTOF (0.05, 0.15), while that of 2 days old was done with TOF (100, 500) and rExtTOF (0.05, 0.15). This filtering is for avoiding the technical noise. |

| 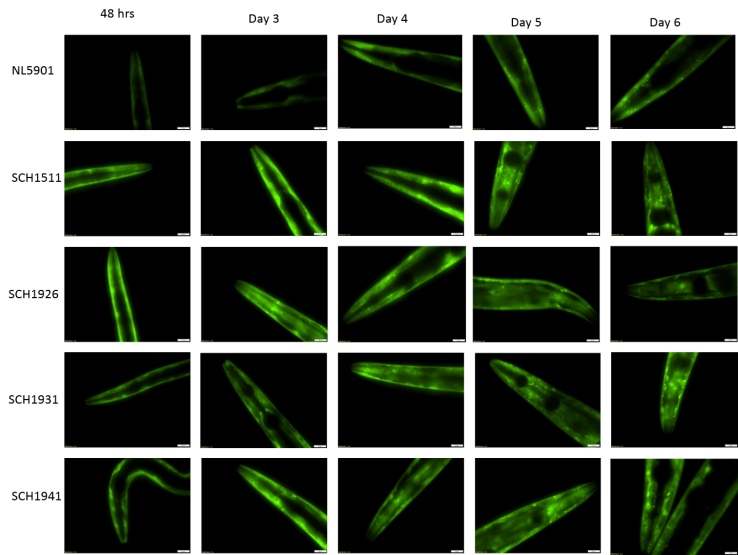A |
| --- |
| **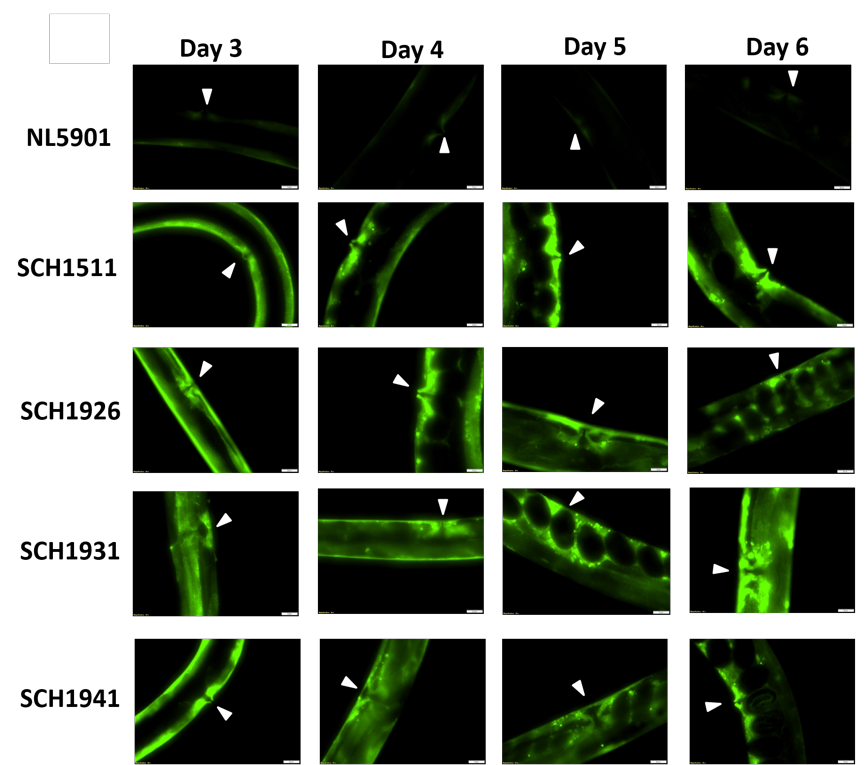B** |
| **Figure S3.** Fluorescent tracking focusing on head area (A) and vulva area (B) over time among the four transgenic ILs and NL5901. From left to right are different ages of worms. Exposure time of all imaging was 600ms. Arrows in B denote the position of the vulva. |
