# Supplementary material for: Genetic Variation in Complex Traits in Transgenic α-Synuclein Strains of Caenorhabditis elegans
Source: Genes (Basel). 2020 Jul 11;11(7):778. doi: 10.3390/genes11070778 (PMC7397059; doi:10.3390/genes11070778)
Supplement: Supplementary file 1 [file genes-11-00778-s001.zip › Short explanation of Table S1-S6.docx]

**Table S1:** **The dataset of YFP measurement obtained by filtering out** **technical noise.** Raw data of all 3 day-old worms, which was collected from a Union Biometrica COPAS BIOSORT large particle flow, were filtered with both TOF (250, 1000) and rExtTOF (0.05, 0.15), while that of 2 day-old was done with TOF (100, 500) and rExtTOF (0.05, 0.15). This filtering is for avoiding the technical noise.

**Table S2: The dataset of Nile Red measurement obtained by filtering out technical noise.** Raw data of all 3 day-old worms, which was collected from a Union Biometrica COPAS BIOSORT large particle flow, were filtered with both TOF (250, 1000) and rExtTOF (0.05, 0.15), while that of 2 day-old was done with TOF (100, 500) and rExtTOF (0.05, 0.15). This filtering is for avoiding the technical noise.

**Table S3: The expression of** **α‑synuclein transgene over all αS-carrying strains (αS ILs and NL5901), including** **two-samples t-test and two-way Anova.** Data are expressed as mean ± standard deviation, and analysed using two-samples t-test, followed by two-way Anova with model lm(mean ~ Primer.inf+Strain).

**Table S4: The quantification of green fluorescent signal over all** **αS-carrying strains (αS ILs and NL5901) based on Table S1, including one-way Anova and Tukey’s test**. Each strain was measured at 2-day- and 3-day-old. Data are expressed as mean ± standard deviation, and analysed using one-way ANOVA with model lm (norm.Green~Strain), followed by Tukey’s test.

**Table S5: The quantification of TOF over all αS-carrying strains (αS ILs and NL5901) and their corresponding wild-type strains based on Table S2 of Nile Red assay, including** **two-way Anova and Tukey’s test.** Each strain was measured at 2-day- and 3-day-old. Data are expressed as mean ± standard deviation, and analysed using two-way ANOVA with model lm(TOF~Genotype+alphasyn), followed by Tukey’s test.

**Table S6:** **The quantification of Nile Red staining over all α‑synuclein transgenic lines and their corresponding wild-type strains based on Table S2, including two-way Anova and Tukey’s test**. Each strain was measured at 2-day- and 3-day-old. Data are expressed as mean ± standard deviation, and analysed using two-way ANOVA with model lm(norm.Red~Genotype+alphasyn), followed by Tukey’s test.
